# Metabolites from nematophagous fungi and nematicidal natural products from fungi as an alternative for biological control. Part I: metabolites from nematophagous ascomycetes

**DOI:** 10.1007/s00253-015-7233-6

**Published:** 2015-12-29

**Authors:** Thomas Degenkolb, Andreas Vilcinskas

**Affiliations:** Institute for Insect Biotechnology, Justus-Liebig-University of Giessen, Heinrich-Buff-Ring 26-32, 35392 Giessen, Germany; Department of Bioresources, Fraunhofer Institute for Molecular Biology and Applied Ecology, Winchester Strasse 2, 35394 Giessen, Germany

**Keywords:** Phytoparasitic nematodes, Nematicides, Oligosporon-type antibiotics, Nematophagous fungi, Secondary metabolites, Biocontrol

## Abstract

Plant-parasitic nematodes are estimated to cause global annual losses of more than US$ 100 billion. The number of registered nematicides has declined substantially over the last 25 years due to concerns about their non-specific mechanisms of action and hence their potential toxicity and likelihood to cause environmental damage. Environmentally beneficial and inexpensive alternatives to chemicals, which do not affect vertebrates, crops, and other non-target organisms, are therefore urgently required. Nematophagous fungi are natural antagonists of nematode parasites, and these offer an ecophysiological source of novel biocontrol strategies. In this first section of a two-part review article, we discuss 83 nematicidal and non-nematicidal primary and secondary metabolites found in nematophagous ascomycetes. Some of these substances exhibit nematicidal activities, namely oligosporon, 4′,5′-dihydrooligosporon, talathermophilins A and B, phomalactone, aurovertins D and F, paeciloxazine, a pyridine carboxylic acid derivative, and leucinostatins. Blumenol A acts as a nematode attractant. Other substances, such as arthrosporols and paganins, play a decisive role in the life cycle of the producers, regulating the formation of reproductive or trapping organs. We conclude by considering the potential applications of these beneficial organisms in plant protection strategies.

## Introduction

### Nematodes as economically important crop pests

Among more than 26,000 known species of nematodes, 8000 are parasites of vertebrates (Hugot et al. 2001), whereas 4100 are parasites of plants, mostly soil-borne root pathogens (Nicol et al. 2011). Approximately 100 species in this latter group are considered economically important phytoparasites of crops. Nematodes can damage all parts of their host plants, although according to their life style, individual species target the stems, leaves, flowers, or seeds. Root nematodes can be subdivided further into free-living ectoparasites, migratory species, and sedentary endoparasites (Dowe 1987). The latter group is responsible for most damage to crops (Jansson and López-Llorca 2004). It includes the root-knot nematodes (*Meloidogyne* spp.) and the cyst nematodes (*Heterodera* and *Globodera* spp.). A characteristic feature of most phytoparasitic nematodes is their protractible stylet, a spear-shaped hollow feeding organ that is used to puncture cells.

Currently, the three most prevalent phytoparasitic nematodes in Europe are the beet cyst nematode *Heterodera schachtii*, the stem and bulb nematode *Ditylenchus dipsaci*, and the golden potato cyst nematode *Globodera rostochiensis*. The latter is a quarantine organism, which was the major potato crop pest until the 1980s when it was brought under control by the development of potato cultivars resistant to the nematode pathotype Ro1. Other invasive cyst nematodes pose a risk if accidentally introduced into Europe because they may outcompete the well-controlled incumbent species. For example, the white potato cyst nematode (*Globodera pallida*) would probably outcompete *G. rostochiensis* pathotype Ro1 after a number of rotations without targeted countermeasures (KWS Saat 2012). Among the 97 validly described *Meloidogyne* species (Hunt and Handoo 2009), the northern root-knot nematode (*Meloidogyne hapla*) is dominant in temperate regions, followed by *Meloidogyne naasi* and *Meloidogyne chitwoodi*, whereas in warmer climates and under glasshouse conditions, crops are predominantly infected with *Meloidogyne fallax*, *Meloidogyne incognita*, *Meloidogyne arenaria*, or *Meloidogyne javanica* (Wesemael et al. 2011).

Nematode damage in crops is non-specific and causes a range of symptoms from mild to severe, such as wilting, stunting, reduced vigor, nutrient deficiency, root lesions, reduced flowering, fruit loss, poor yield, and even death. Mild symptoms may be overlooked, and even the severe symptoms can be misdiagnosed (Nicol et al. 2011). Only few diagnostic symptoms are apparent. Above ground, these include small chlorotic or necrotic patches that spread rapidly from the site of infection. Below ground, they include root galls, cysts, and clumps of nematodes that are commonly described as “nematode wool.” Worldwide annual yield losses caused by phytoparasitic nematodes are 3.3–20.6 %, averaging 12.3 %. Losses in the tropics and subtropics are generally higher than in temperate zones, and developing countries are more severely affected than industrialized countries. The global financial impact of phytoparasitic nematodes may be as high as US$ 121 billion, including US$ 9.1 billion in the USA (Chitwood 2003; Nicol et al. 2011).

### Chemical control of phytoparasitic nematodes

Nematicidal chemicals for crop protection must be sufficiently volatile and water-soluble to ensure an even distribution in the upper soil layer without absorption to soil particles. This requires high doses during application, which contradicts groundwater protection policies. Even if these physical barriers are overcome, the chemical control of economically important cyst nematodes is remarkably inefficient because most nematicides cannot penetrate the proteinaceous matrix of the hardened cyst. Phytoparasitic nematodes reproduce at a prodigious rate, so the overall effectiveness of many classical nematicides is questionable.

Both registered and obsolete nematicides (including soil fumigants) applied in the USA have been comprehensively reviewed by Chitwood (2003). Many of the classical fumigant nematicides have been banned by the US Environmental Protection Agency (EPA) and/or the European Commission, and the number of non-fumigant nematicides in current use has declined over the last 25 years. In both cases, this reflects concerns about their non-specific mechanisms of action and therefore the potential negative impact on human health and the environment.

By 2015, the broad-spectrum, systemic organothiophosphate fosthiazate (Nemathorin 10 G^®^) was the only nematicide still registered in Germany for the control of larval and adult stages of potato cyst nematodes (*G. rostochiensis* and *G. pallida*) in late season potato varieties. Root-knot nematodes (*Meloidogyne* spp.) as well as migratory endoparasitic root-lesion nematodes (*Pratylenchus* spp.) and stubby-root nematodes (*Trichodorus* spp.) may also be controlled by this chemical. This compound has several desirable side effects, including the reduction in damage to potato tubers caused by wireworms (*Agriotes* spp.), the larvae of click beetles (*Coleoptera*: *Elateridae*). In order to guarantee high levels of safety and efficacy, 3 kg of the active product per hectare (=30 kg of granules) must be incorporated immediately and completely to a soil depth of 10–15 cm using a granule applicator. No spilled granules must remain on the soil surface because fosthiazate is toxic to birds and aquatic organisms (Börner 2009). The product is not registered in Austria or Switzerland because both countries still use the dithiocarbamate dazomet (Basamid^®^), which is hydrolyzed to produce hydrogen sulfide, formaldehyde, methylamine, and methyl isothiocyanate (MITC). The latter compound, which is regarded as the bioactive principle of the precursor dazomet, is nematicidal, insecticidal, herbicidal, and fungicidal. However, it is also toxic to fish and other aquatic organisms, and in humans, it is a lachrymator and a topical and respiratory irritant, eventually causing respiratory edema (EFSA 2010; Römpp 2015).

### Nematophagous fungi for the control of phytoparasitic nematodes

Environmentally beneficial and low-cost alternatives to chemical control measures for phytoparasitic nematodes are needed, and these must not affect vertebrates, crops, and other non-target organisms. Highly specific, preferably soil-borne antagonists are best suited for this purpose. Several entomopathogenic and fungicolous fungi have been registered for the biocontrol of insect pests and soil-borne plant pathogens in Austria, Switzerland, and Germany (AGES 2015; BLW 2015; BVL 2015); therefore, nematophagous fungi could fulfill an analogous role for the biocontrol of phytoparasitic nematodes. Approximately 700 species of nematophagous fungi have been described thus far (Yu et al. 2014), and four ecophysiological categories have been proposed (Yang and Zhang 2014), which are considered in turn below. However, taxonomic identification of the nematode species to be controlled is crucial to achieve a high level of control. In this context, it is important to consider that phytophagous nematodes carrying a stylet are unable to swallow fungal spores, whereas saprotrophic microbiophagous nematodes can take up spores orally (Dowe 1987). This distinction greatly affects the choice of the biocontrol agent to be applied.

#### Nematode-trapping or predacious fungi

form adhesive or mechanical hyphal traps. Earlier classifications based on morphological features such as the architecture of conidiophores; the presence of denticles; and the size, shape, septation, and ornamentation of conidia (Cooke and Godfrey 1964; Haard 1968) are not sufficient for genus and species delimitation. More recent studies have established a phylogenetic link between the trapping devices used by each species (Rubner 1996; Pfister 1997; Ahrén et al. 1998), and the affiliation of most of these “predaceous” fungi to the *Orbiliomycetes* (*Ascomycota*) where they form a monophyletic group (Yang et al. 2007, 2012). Scholler et al. (1999) was the first to draw taxonomic consequences from this by assigning generic rank to groups characterized by different trapping structures, distinguishing four genera and *Dactylella* for related but non-capturing species. Yu et al. (2014) proposed a reclassification of three genera, namely *Arthrobotrys* (54 species), *Drechslerella* (14 species), and *Dactylellina* (28 species). The fourth genus of Scholler et al. (1999), *Gamsylella*, was questioned by Li et al. (2005) in their analysis of 28S and 5.8S rRNA and *β*-tubulin. However, this is still a matter of debate, because the *Gamsylella* species are moderately distinct also at the molecular level.

Among the predaceous *Ascomycota*, five types of trapping device can be distinguished, four of which act passively: (i) adhesive three-dimensional networks; (ii) adhesive unicellular knobs either directly attached to the hyphae or on erect stalks; (iii) non-constricting mostly three-celled rings, which often co-occur with adhesive knobs; and (iv) adhesive columns consisting of a few inflated cells on each hypha. The latter type is also found in nematode-trapping genera outside the *Ascomycota* such as *Stylopage* spp. and *Cystopage* spp. (*Zoopagales*, *Zoopagaceae*). The fifth and most sophisticated type is the constricting ring trap, which is exclusively found in the genus *Drechslerella* and captures nematodes actively. When a nematode enters such a trap, the three cells composing the ring swell in a tenth of a second to close the lumen and immobilize the prey. Notably, slowly growing species may form their trapping organs without stimulation, whereas those showing faster saprotrophic growth, mainly *Arthrobotrys* spp. (Cooke 1968), require external stimuli to induce trap formation. Under natural conditions, the presence of nematodes or nematode secretions (see discussion of paganins A and B below) triggers the formation of these traps (Nordbring-Hertz et al. 2011).

#### Endoparasitic fungi

infect their nematode hosts either via oral uptake or the adhesion of spores or zoospores to the host cuticle. Most of the approximately 50 species of endoparasites are obligate parasites with a broad host range. Once a host has been infected by an endoparasite, the fungus completes its vegetative development within an individual nematode. Endoparasites among the *Ophiocordycipitaceae* attack nematodes either by oral uptake of non-adhesive spores, which germinate in the alimentary tract (*Harposporium* spp.), or adhesion of large numbers of conidia to the surface of the hosts (*Ophiocordyceps* spp*.*, now including *Hirsutella* spp. and *Drechmeria* spp., now including *Haptocillium* spp.^1^). Infective zoospores of *Catenaria anguillulae* (*Blastocladiales*, *Catenariaceae*) encyst after physical contact with the nematode is made and adhere to its cuticle. Upon physical contact, the genus *Haptoglossa* (*Haptoglossales*, *Haptoglossaceae*) violently injects immotile tertiary spores into the body of as nematode (Dowe 1987; Nordbring-Hertz et al. 2011). Still far, too little is known about the biocontrol potential of this group; however, it also appears that the host spectrum of some endoparasites is rather limited. The conidia of *Drechmeria coniospora*, for instance, have been shown to adhere to bacteriovorous, phyto-, and animal-parasitc nematodes. However, this adhesion did not necessarily led to penetration and subsequent infection of the nematodes tested (Jansson 1993).

#### Nematode egg and female parasites

target the immobile stages of economically important genera of cyst and root-knot nematodes such as *Globodera*, *Heterodera*, *Rotylenchulus*, *Tylenchulus*, and *Meloidogyne*. Cysts can persist in the soil for several years, so older ones may be colonized by multiple nematophagous and non-nematophagous fungal species.

The three-layer shell of nematode eggs is another target attacked by opportunistically parasitic nematophagous fungi. Most of them use appressoria to mechanically penetrate host eggs (López-Llorca et al. 2008). In some cases, penetration is facilitated by extracellular enzymes such as chitinases and proteases; thus, parasitism by efficient enzyme producers is more prominent (Yang et al. 2007).

#### Toxin-producing fungi

attack their prey by secreting diffusible toxins, and this takes place before any physical contact between the fungus and nematode (López-Llorca et al. 2008). This artificial group comprises basidiomycete genera such as *Pleurotus* and *Coprinus*, but also ascomycetes that clearly belong to one of the three aforementioned groups.

## Metabolites produced by nematophagous fungi

### Overview

The chemical ecology of nematophagous fungi is still far from understood. Little has been done to screen for metabolites in nematophagous fungi, or nematicidal metabolites in other fungi, since the pioneering studies by Stadler and colleagues published in the 1990s (Stadler et al. 1993a, b, 1994). The first part of this review article discusses the known metabolites from nematophagous ascomycetes, whereas the second part will focus on nematicidal metabolites from nematophagous basidiomycetes and non-nematophagous fungi.

### Metabolites from nematode-trapping ascomycetes

**Fig. 1:**
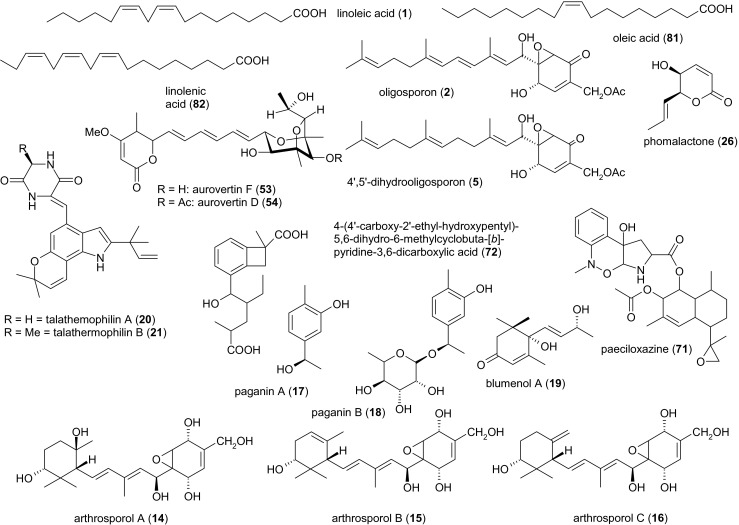
Secondary metabolites of nematode-trapping, female-, and egg-parasitic fungi, which either display nematicidal (**1**, **2**, **5**, **20**, **21**, **23**, **53**, **54**, **71**, **72**, **81**, **82**) or nematode-attracting activities (**19**). Compounds (**14**–**18**) auto-regulate the morphology of the producing fungus

**Fig. 2:**
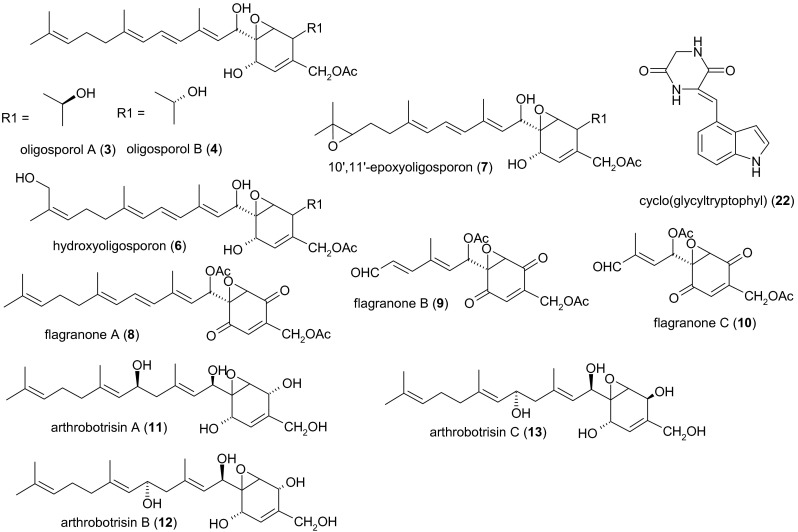
Other secondary metabolites from nematode-trapping fungi

Secondary metabolites from nematode-trapping ascomycetes, which either display nematicidal and nematode-attracting activities or regulate the formation of reproductive or trapping organs, are summarized in Fig. 1. Other secondary metabolites from nematode-trapping fungi are depicted in Fig. 2.

Linoleic acid [(9*Z*,12*Z*)-octadeca-9,12-dienoic acid] (compound **1**) is regarded as the only bioactive principle in many nematode-trapping fungi. It was originally obtained from submerged cultures of *Arthrobotrys conoides* Bit 1. The lethal dose (LD)_50_ against the model nematode *Caenorhabditis elegans* was determined to be 5–10 μg/ml, compared to 0.1 μg/ml for the antihelminthic standard ivermectin. Notably, the number of three-dimensional traps formed by the fungus was positively correlated with the amount of linoleic acid produced (Stadler et al. 1993a). Nematicidal amounts of linoleic acid have also been reported in *Arthrobotrys oligospora*, *Drechslerella brochopaga*, and *Drechslerella dactyloides* (Stadler et al. 1993b).

Metabolites from *A. oligospora* CBS 115.81 include the three colorless oils: oligosporon, oligosporol A, and oligosporol B (compounds **2**–**4**). The antimicrobial activity of the culture filtrate was detected after 55 h, but the fermentation was continued until the glucose in the medium was used up after 144 h. The formation of compounds **2**–**4** was also observed on other media and under different fermentation conditions. *A. conoides*, *D.* (syn. *Dactylella*) *brochopaga*, and *D*. (syns. *Arthrobotrys*/*Dactylaria*) *dactyloides* were also assigned as producers of these compounds. Among a number of bacteria and yeasts used as test organisms, only *Bacillus brevis* and *Bacillus subtilis* displayed moderate susceptibility. All three compounds also exhibited moderately cytotoxic and hemolytic effects, but no phytotoxic or mutagenic activity. The compounds are not nematicidal against *C. elegans* (Stadler et al. 1993b), but the LD_50_ of oligosporon against larvae of the nematode *Haemonchus contortus*, which infects ruminants, was subsequently found to be 25 μg/ml (Anderson et al. 1995).

Three further oligosporon-type metabolites from *A. oligospora* M2 were identified as (4*S*,5*R*,6*R*)-4′,5′-dihydrooligosporon, (4*S*,5*R*,6*R*)-hydroxyoligosporon, and (4*S*,5*R*,6*R*)-10′11′-epoxyoligosporon (compounds **5**–**7**). These were obtained from 7-day-old shaken cultures as three colorless oils. The same strain also produced compounds **2**, (4*S*,5*R*,6*R*)-oligosporon, and **4**, both of which displayed weak antibiotic activity against *B. subtilis*. Compound **4** weakly inhibited *Streptomyces aureofaciens* whereas compound **2** weakly inhibited the root rot pathogen *Phytophthora cinnamomi*. No other activities against Gram-negative bacteria or fungi were observed. Compound **5** was the only new compound to display a weak nematicidal activity (LD_50_ = 50–100 μg/ml) against *H. contortus* (Anderson et al. 1995).

Flagranones A, B, and C (compounds **8**–**10**) were produced by a strain of *Arthrobotrys flagrans* (syn. *Duddingtonia flagrans*) isolated from cattle feces and cultivated by solid-state fermentation on wheat, oats, and barley grains. After 4 weeks, the grains were combined and extracted, and three yellow oils were obtained that are structurally related to oligosporon (**2**), suggesting a common biosynthetic metabolic origin. Flagranones A and B were active against *B. subtilis* and *S. aureofaciens* with a minimal inhibitory concentration (MIC) of 25 μg/ml. Gram-negative bacteria such as *Escherichia coli* and *Erwinia carotovora* were weakly inhibited, and the phytopathogenic fungi *P. cinnamomi*, *Pythium ultimum*, and *Rhizoctonia solani* were partially inhibited. Nematicidal activity in the *H. contortus* larval development assay was classified as insignificant (Anderson et al. 1999).

Arthrobotrisins A, B, and C (compounds **11**–**13**) are oligosporon-type antibiotics obtained as colorless oils from submerged cultures of *A. oligospora* YMF 1.3170 after cultivation for 10 days. Strong activity was observed against *B. subtilis*, but the activity against two other *Bacillus* species and *Staphylococcus aureus* were moderate in comparison to the bacteriostatic standard chloramphenicol. None of the arthrobotrisins displayed nematicidal activity against the free-living nematode *Panagrellus redivivus* (Wei et al. 2011).

Arthrosporols A, B, and C (compounds **14**–**16**) are additional oligosporon-type metabolites carrying a 1,3,3-trimethylcyclohexyl ring instead of a farnesyl side chain. They were obtained as amorphous solids from *A. oligospora* YMF 1.3170 after 4 days of submerged cultivation at 28 °C. All three substances play an important role in the regulation of fungal morphology. Concentrations of 0.5–5 μg/ml inhibit the formation of conidiophores, with compound **16** exhibiting the strongest effect (50–90 % inhibition). Compound **16** (at 5 μg/ml) also inhibited conidial germination within 4 h by 43 %. Notably, compounds **14** and **16** triggered the formation of trapping organs, i.e., a three-dimensional network (Zhang et al. 2012). A recent HPLC analysis revealed that arthrosporol A could not be detected anymore after disruption of the polyketide synthase (PKS) gene *AOL_s00215g283*, which encodes for the biosynthesis of 6-methylsalicylic acid in *A. oligospora* YMF 1.3170. In comparison to the wild-type strain, the mutant *ΔAOL_s00215g283* showed a tenfold increase in the number of traps formed. The number of nematodes captured by the mutant strain was more than twice as high as in the wild type (Xu et al. 2015). However, a detailed LC-MS/MS-based study is urgently required to unequivocally characterize the metabolic changes observed. All oligosporon-type antibiotics known to date (compounds **2**–**16**) seem to be of mixed biosynthetic origin; however, no studies addressing this question have been published yet. Obviously, the carbon skeleton is formed by alkylation of a polyketide-derived 7-oxabicyclo[4.1.0]hept-3-ene nucleus, whereas the farnesyl side chain originates from isoprenoid precursors (Anderson et al. 1995).

Paganins and other metabolites have also been isolated from *Dactylellina entomopaga* CBS 642.80.^2^ Paganins A and B (compounds **17** and **18**), 1,2,4-trisubstituted simple phenols, were isolated as two colorless oils from 14-day-old shaken cultures of *D. entomopaga* (syn. *Arthrobotrys entomopaga*). As previously observed for compounds **14** and **16**, both paganins inhibited the formation of conidiophores (25 μM of each compound resulted in a 65–75 % reduction). Both compounds slightly inhibited the formation of adhesive knobs (trapping organs) at a concentration of 5 μm, but 50 μM of compound **17** increased the formation of adhesive knobs by 118 %. Spore germination was also inhibited by compounds 17 and 18 (55–75 % inhibition within 12 h in the presence of 50 μm compound **17**, and 40–45 % inhibition within 8 h in the presence of 50 μm compound **18**). Compound **17** and the simultaneously isolated compound **19** (blumenol A, a so-called bis-nor-isoprenoid of uncertain biosynthetic origin^3^) were strong attractants for *C. elegans*, whereas compound **18**, in which the side chain is substituted with an *α*-6-deoxy-l-mannopyranosyl residue, showed no such effect. Neither of the compounds inhibited *B. subtilis* or showed nematicidal activity against *P. redivivus* (Wu et al. 2013). *D. entomopaga* also produces talathermophilins A and B (compounds **20** and **21**), two yellow pyranoindole alkaloids with prenylated side chains.^4^ Although these compounds initially appeared to show no nematicidal activity, 400 μg/ml was subsequently shown to inhibit the free-living nematode *P. redivivus* by 38 % (compound **20**) and 44 % (compound **21**), respectively (Chu et al. 2010). Finally, no bioactive properties of the diketopiperazine cyclo(glycyltryptophyl) (compound **22**) have been reported thus far (Guo et al. 2011; Wu et al. 2013).

### Metabolites from fungi that infect eggs and cysts

**Fig. 3:**
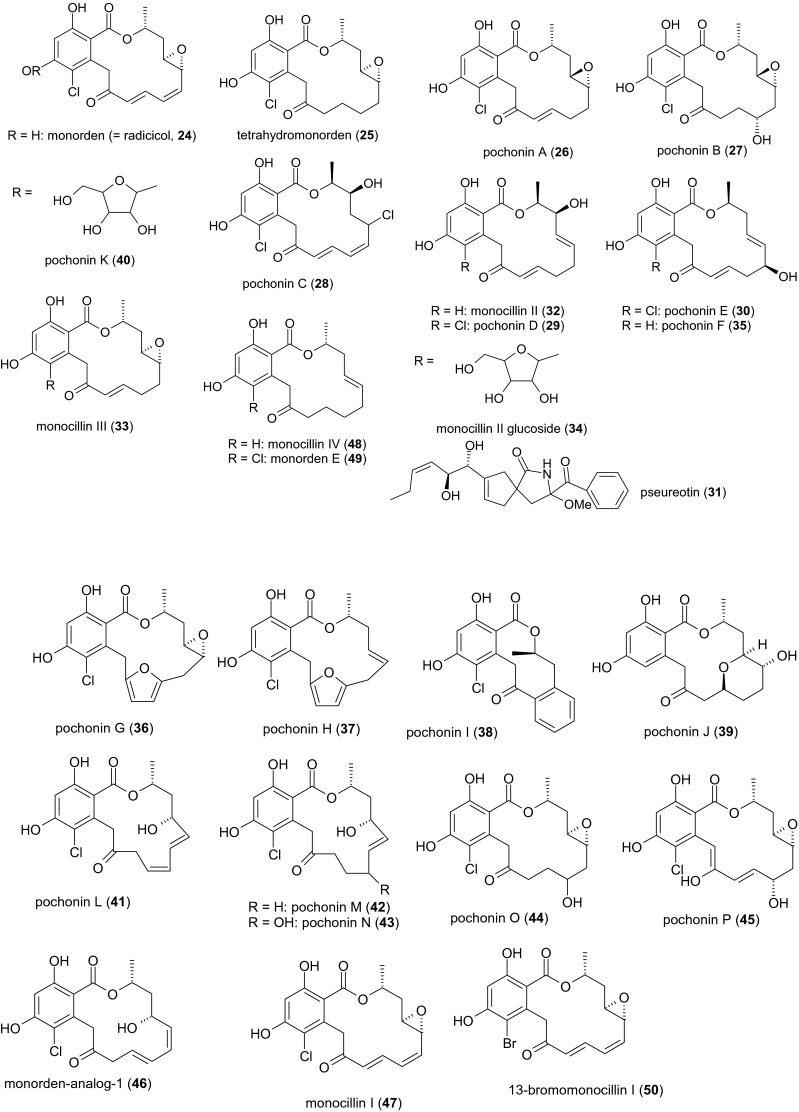

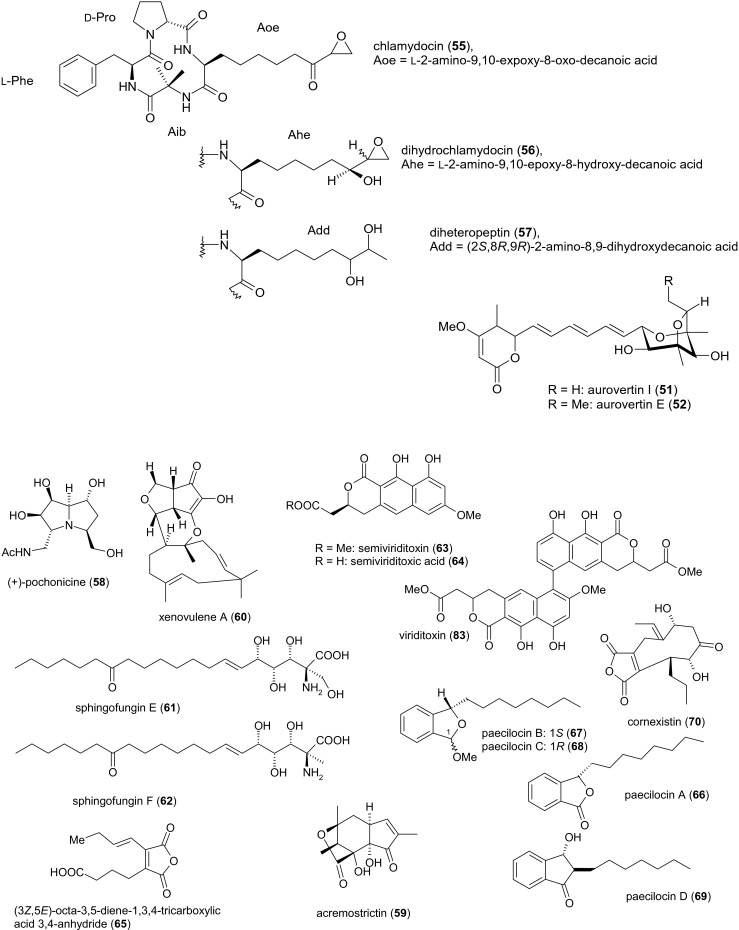
Other secondary metabolites from female- and egg-parasitic fungi

Secondary metabolites from fugal pathogens of female nematodes and nematode eggs, which display nematicidal activities, are summarized in Fig. 1. Other secondary metabolites from this group are shown in Fig. 3. Peptaibiotics, including the nematicidal leucinostatins produced by members of this group, are listed in Table 1.

**Table 1:** Sequences of nematicidal, non-ribosomally biosynthesized peptide antibiotics (peptaibiotics) from *Paecilomyces variotii* (**73**) and *Purpureocillium lilacinum* (**75**–**80**)

No.	Residue																					
		1	2	3	4	5	6	7	8	9	10	11	12	13	14	15	16	17	18	19	20	21
**73**	MOTDA	Pro	Aib	Aib	Aib	Aib	Ala	Ala	Aib	Leu	Ala	Aib	Ala	Ala	Aib	Arg	Ala	Aib	Gly	Aib	Aib	Ala
**75**	MeHA	MePro	AHMOD	Hyleu	Aib	Leu	Leu	Aib	Aib	*β*-Ala	DPD											
**76**	MeHA	MePro	Leu	Hyleu	Aib	Leu	Leu	Aib	Aib	*β*-Ala	DPD											
**77**	MeHA	MePro	Leu	Hyleu	Aib	Leu	Leu	Aib	Aib	*β*-Ala	MPD											
**78**	MeHA	MePro	Leu	Hyleu	Aib	Leu	Leu	Aib	Aib	*β*-Ala	DPD-NO											
**79**	MeHA	Pro	AHMOD	Hyleu	Aib	Leu	Leu	Aib	Aib	*β*-Ala	MPD											
**80**	MeHA	Pro	Leu	Hyleu	Aib	Leu	Leu	Aib	Aib	*β*-Ala	MPD											

*MOTDA β*-keto-2-methyltetradecanoic acid, *MeHA* (4*S*,2*E*)-4-methylhex-2-enoic acid, *MePro cis*-4-l-methylproline, *AHMOD* (2*S*,4*S*)-2-amino-6-hydroxy-4-methyl-8-oxodecanoic acid, *Aib* α-aminoisobutyric acid, *Hyleu threo*-*β*-l-hydroxyleucine, *β*-*Ala β*-alanine, *MPD N* ^1^-methyl-propane-1,2-diamine, *DPD N* ^1^ *N* ^1^-dimethyl-propane-1,2-diamine, *DPD-NO* (2*S*)-*N* ^1^,*N* ^1^-dimethyl-propane-1,2-diamine-*N*-oxide

Phomalactone (compound **23**) from *Pochonia chlamydosporia* was previously isolated from *Phoma* sp., *Drechslera* sp., *Curvularia inaequalis* (*Pleosporales*) and *Nigrospora* sp. (*Trichosphaeriales*). It was obtained as a colorless liquid from 14-day-old submerged cultures of strain VC10 (syns. *Diheterospora chlamydosporia* and *Verticillium chlamydosporium*). Phomalactone at a concentration of 500 mg/l exhibited promising inhibitory activity against the second-stage larvae of *M. incognita* in a tomato root invasion assay, comparable to 100 mg/l of aldicarb standard. However, phomalactone was less toxic than commercial aldicarb and oxamyl standards, and its lethal effect was delayed in the *M. incognita* mortality assay. The co-isolated major compound monorden (compound **24**) showed no activity in either assay (Khambay et al. 2000).

Macrocyclic polyketides containing a 2,4-dihydroxybenzoate (resorcylate) residue attached to a 14-membered lactone ring system are usually referred to as resorcylic acid lactones (RALs). Biosynthesis of radicicol (displaying d-configuration at C10′)^5^ has recently been shown to be catalyzed by two iterative polyketide synthases (PKS), one of which is a highly reducing (hrPKS) and the other a non-reducing PKS (nrPKS). Subsequent macrocyclization is mediated by an stereotolerant thioesterase, which is capable of generating the native d- and the enantiomeric l-macrocycle (Heberlig et al. 2014). A number of RALs and an alkaloid were isolated from *P. chlamydosporia* var. *catenulata* P 0297 (syn. *V. chlamydosporium* var. *catenulatum*). Fermentation for 168 h at 23 °C in chloride-containing MGP medium led to the isolation of monorden^6^ (compound **24**) and its derivative tetrahydromonorden (compound **25**) as well as five structurally related compounds named pochonins A–E (compounds **26**–**30**) and the spirocyclic alkaloid pseurotin^7^ (compound **31**). The replacement of chloride in the medium with bromide resulted in the isolation of monorden (compound **24**); the two major metabolites, monocillins II and III (compounds **32** and **33**); and two minor compounds, monocillin II glucoside (compound **34**) and pochonin F (compound **35**). Whereas monocillins I–III were isolated as colorless solids, monocillin IV and the pochonins were isolated as brownish or colorless oils. Most of these compounds were moderately active against *Herpes simplex* virus type I (HSV-1) and *Eimeria tenella*, the agent responsible for hemorrhagic cecal coccidiosis in poultry. They were also cytotoxic. The remarkably broad spectrum of biological activities previously reported for monorden (compound **24**), which has previously been isolated from *Monocillium nordinii* (*Niessliaceae*, *Hypocreales*)^8^ and other fungi, was also demonstrated. The native role of compounds **24**–**35** is unclear because they did not modulate the activity of the human estrogenic receptor ER*β* (Hellwig et al. 2003) unlike zearalenone and its derivatives (Frisvad et al. 2006).

Additional RALs were isolated from *P. chlamydosporia* var. *chlamydosporia* FERM BP-8332 (strain TF-0480) during a screen for WNT-5a inhibitors (Shinonaga et al. 2009a). This strain produced monorden (compound **24**) and four novel RALs named pochonins G–J (compounds **36**–**39**). A more detailed investigation of the same strain yielded four known RALs, i.e., pochonins B, D, E, and F (compounds **27**, **29**, **30**, and **35**) as well as the novel pochonins K–P (compounds **40**–**45**). Further RALs from the same fermentation comprised monorden analog-1 (compound **46**) and monocillins I–IV (compounds **47**, **32**, **33,** and **48**) as well as monorden E (compound **49**) (Shinonaga et al. 2009b). When the growth medium was supplemented with sodium bromide, the fermentation also yielded 13-bromomonocillin (compound **50**) (Shinonaga et al. 2009c).

Four of the 19 aurovertins (A–S) known to date were retrieved from *P. chlamydosporia* YMF 1.00613 by passing infected *M. incognita* juveniles from tobacco root knots through a Baermann funnel and cultivating the fungi in submerged culture for 12 days at 28 °C. This resulted in the recovery of the novel compound aurovertin I (compound **51**) as well as three other yellow oils representing the known aurovertins E, F, and D (compounds **52**–**54**). After 48 h, the major aurovertins D and F displayed nematicidal activity towards the free-living species *P. redivivus* with LC_50_ values of 41.7 and 88.6 μg/ml, respectively. However, the four aurovertins were unable to prevent *M. incognita* eggs from hatching at concentrations of 25–400 μg/ml, and the inhibitory efficacy was below 5 % (Niu et al. 2010). In a recent study, aurovertin D (compound **54**) was strongly toxic towards *M. incognita* and affected *C. elegans* even at sub-inhibitory concentrations. DAF-2, an evolutionary conserved insulin/IGF-1-like receptor, is required for timing of larval development in *C. elegans*, whereas DAF-16 is a *C. elegans* FOXO transcription factor acting as a key mediator for multiple stress responses. Consequently, resistance of *C. elegans daf*-*2* (*e1370*) and hypersensitivity of *C. elegans daf*-*16* (*mu86*) to aurovertin D indicated that DAF-16/FOXO transcription factor in nematodes was triggered in response to aurovertin (Wang et al. 2015). The 2,6-dioxabicyclo[3.2.1]octane (DBO) ring system found throughout the aurovertin family. Briefly, a hexaene pyrone is biosynthesized by highly reducing PKS, AurA. Next, the OH-group of this pyrone is methylated by an *O*-methyltransferase. A flavin-dependent monooxygenase and an epoxide hydrolase convert the triene side chain into the dioxabicyclo[3.2.1]octane scaffold (Mao et al. 2015).

The interconvertible cyclotetrapetaibiotics (Degenkolb et al. 2008) chlamydocin (compound **55**) and dihydrochlamydocin (compound **56**) were obtained from *P. chlamydosporia* NRRL 3472 culture filtrates after fermentation for 90 h. Pure chlamydocin appears as white foam, whereas dihydrochlamydocin forms white crystals. Both substances showed strong cytostatic activity towards mastocytoma cells (Closse and Huguenin 1974) and inhibited histone deacetylases (Islam et al. 2012). Another cyclotetrapetaibiotic,^9^ diheteropeptin (compound **57**), was obtained as a white powder from submerged cultures of *P. chlamydosporia* Q58044 after 5 days. This metabolite mimics the activity of transforming growth factor beta (TGF*β*) and inhibits histone deacetylase (Masuoka et al. 1997, 2000a, b). These three compounds have yet to be tested for their nematicidal effects.

The polyhydroxylated pyrrolizidine alkaloid pochonicine (compound **58**) was isolated from *Pochonia suchlasporia* var*. suchlasporia* TAMA 87 as colorless syrup during solid-state fermentation. This compound is a potent inhibitor of *β*-*N*-acetylglucosaminidases from diverse sources, including fungi, insects, mammals, and plants (Usuki et al. 2009). The structure of pochonicine was recently revised to be (+)-(1*R*,3*S*,5*R*,6*R*,7*S*,7a*R*)-pochonicine (Zhu et al. 2013).

The tricyclic lactone acremostrictin (compound **59**) was recently isolated from the polyphagous species *Acremonium* (now *Sarocladium*) *strictum*^10^ MB05005,^11^ which originated from an unidentified *Choristida* sponge. *S. strictum* is known as an aggressive parasite of *H. schachtii* eggs (Nigh et al. 1980). The compound was isolated from the filtrate of a 3-week-old submerged culture of a marine isolate. It shows weak antibacterial and moderate antioxidant activities, but additional effects have not been reported thus far (Julianti et al. 2011).

The oxygenated humulene-derived sesquiterpene xenovulene A (**60**) carries an 11-membered ring system, which probably originates from polyketide synthesis. It was isolated from the mycelium of a 5-day-old fermentation of *A. strictum* IMI 354451. The compound inhibits the binding of flunitrazepam to the benzodiazepine site of the *γ*-aminobutyric acid (GABA)_A_ receptor with an IC_50_ value of 40 nM (Ainsworth et al. 1995).

Two sphingolipids, sphingofungins E and F (compounds **61** and **62**), were isolated from a 14-day-old solid-state fermentation of *Paecilomyces variotii* ATCC 74097,^12^ a species that parasitizes the eggs of the soybean cyst nematode *Heterodera glycines* among others (Dowe 1987). Both compounds are sphingosine-like inhibitors of serinepalmitoyl transferase. Sphingofungin E (compound **61**) showed moderate antifungal activity, whereas sphingofungin F (compound **62**) was comparatively weak (Horn et al. 1992).

The entomopathogenic fungal strain *P. variotii* UAMH 6353 isolated from larvae of the mountain pine beetle *Dendroctonus ponderosae* has been shown to produce two polyketide metabolites derived from 1*H*-naphtho[2,3c]pyran-1-one (naphthopyranone). The mycelium of a 3-week-old surface culture produced (*S*)-semi-viriditoxin (compound **63**) and (*S*)-semi-viriditoxic acid (**64**) was present in the mycelium and culture filtrate. Both metabolites exhibited weak antibacterial but no antifungal activity (Ayer et al. 1991).

A tricarboxylic acid anhydride has been isolated from a 5-day-old fermentation of *P. variotii* ICI No. 3142. The culture filtrate contained (*3Z,5E*)-octa-3,5-diene-1,3,4-tricarboxylic acid 3,4-anhydride (compound **65**) (Aldridge et al. 1980) .

Four polyketides named paecilocins A–D (compounds **66**–**69**) were obtained from *P. variotii* J08NF-1, isolated as a symbiont of the jellyfish *Nemopilema nomurai*. Paecilocin A, also known as (*S*)-7-hydroxy-3-octylphthalide or (3*S*)-7-hydroxy-3-octyl-2-benzofuran-1(3*H*)-one, appears as a white amorphous powder, whereas paecilocins B ((1*S*,3*S*)-1,3-dimethoxy-1-octyl-1,3-dihydro-2-benzofuran-4-ol), C ((1*S*,3*R*)-1,3-dimethoxy-1-octyl-3H-2-benzofuran-4-ol) and D ((2*R*,3*R*)-2-heptyl-3,4-dihydroxyindan-1-one) are yellow oils. Compounds **66**–**68** displayed remarkable antibacterial activity (MICs ranging from 5 to 40 μg/ml) against *S. aureus*, including the methicillin-resistant strain 3089 and the multidrug-resistant *Vibrio parahaemolyticus* 7001 (Liu et al. 2011).

Cornexistin ((*4*R,5*R*,8*R*)-9-ethylidene-4,5,7,8,9,10-hexahydro-5,8-dihydroxy-4-propyl-1*H*-cyclonona[c]furan-1,3,6-trione; compound **70**) was isolated as colorless crystals from *P. variotii* strain ATCC 74286 (= FERM BP-1351 = SANK 21086) sourced from deer dung, during a screen for microbial secondary metabolites with herbicidal activity (Nakajima et al. 1991). The maximum concentration of this nonadride^13^ was achieved after 161 h of fermentation at 26 °C. Although cornexistin lacks antimicrobial activity, it showed strong post-emergence herbicidal activity against young annual and perennial monocotyledonous and dicotyledonous weeds but no activity against maize plants.

The pyrrolobenzoxazine paeciloxazine (compound **71**) was isolated from submerged cultures of *Paecilomyces* sp. BAUA3058 after 120 h at 25 °C, during a screen for novel nematicidal metabolites. After 4 days of incubation, the MIC against the nematode *Rhabditella axei* (syn. *Rhabditis pseudoelongata*) was 50 μg/ml, compared to 0.125 μg/ml for the standard ivermectin. Paeciloxazine also showed insecticidal activity towards *Culex pipiens pallens* L_2_ larvae, with a MIC of 25 μg/ml after 24 h, 40-fold lower than the standard imidacloprid. The LD_50_ against *Plutella xylostella* L_3_ larvae was 250 μg/ml (Kanai et al. 2004).

A nematicidal metabolite has also been isolated from *Paecilomyces* sp. YMF 1.0716. Extracts from 40 strains of *Paecilomyces* spp. cultivated for 15 days at 25 °C were tested for nematicidal activity, and the most active extract yielded the white crystalline compound, 4-(4′-carboxy-2′-ethyl-hydroxypentyl)-5,6-dihydro-6-methylcyclobuta[b]pyridine-3,6-dicarboxylic acid (compound **72**). LD_50_ values were determined against three nematodes: *P. redivivus* (52.4 μg/ml), *M. incognita* (47.1 μg/ml), and *Bursaphelenchus xylophilus* (167.7 μg/ml) (Liu et al. 2009).

Two antifungal peptaibiotics were isolated from the culture filtrate of *P. variotii* SCF 1559 after 24 h, the 21-residue peptide SCH 643432 (compound **73**) and a structurally unresolved positional isomer (compound **74**, not shown). Both compounds were active against *Candida albicans* and other *Candida* species as well as dermatophytes of the genus *Trichophyton* (Hegde et al. 2003).

Thus far, 25 leucinostatins have been isolated from various strains of *Purpureocillium lilacinum*^14^ (Fukushima et al. 1983a, b; Isogai et al. 1992; Martinez and Moraes 2015). Although these lipoaminopeptides, a subclass of membrane-active peptaibiotics, have been known since 1973 (Degenkolb and Brückner 2008), their nematicidal activity against *C. elegans* was not reported until Park et al. (2004) investigated the properties of 20 Australian isolates of *P. lilacinum*. The three most potent isolates were shown to produce a mixture of leucinostatins, among which leucinostatins B, D, F, H, L, and T could be identified (compounds **75**–**80**). When tested against a mixed population of *C. elegans* juveniles and adults, this leucinostatin mixture achieved 77 % mortality after 2 h and 100 % mortality after 12 h, at an overall concentration of 100 μg/ml, and 74 % mortality after 24 h at an overall concentration of 10 μg/ml. Egg hatching was not influenced by leucinostatins, but the 100 μg/ml solution killed 100 % of *C. elegans* L_2_ larvae after 24 h, and the 10 μg/ml solution killed 78 % of the L_2_ larvae after 24 h. Five other leucinostatin-negative strains were shown to produce oleic acid, also known as (9*Z*)-octadecenoic acid (compound **81**), linoleic acid (compound **1**), and linolenic acid, also known as (9Z,12*Z*,15*Z*)-octadeca-9,12,15-trienoic acid (compound **82**) (Park et al. 2004), all of which have nematicidal activity (Stadler et al. 1993a, 1994). The nematicidal activity of these strains is also enhanced by their secretion of chitinases (Park et al. 2004). Sequences of linear peptaibiotics reviewed here are listed in Table 1.

### Outlook and perspectives

Among the approximately 30,000 known natural products from fungi, 15,000–16,000 are considered to be bioactive and almost 12,000 of them have been isolated from microscopic fungi (Bérdy 2012). For example, the beneficial role of antifungal peptidic and non-peptidic secondary metabolites in commercial biocontrol agents formulated with species of the *Trichoderma harzianum* complex (Chaverri et al. 2015) has recently been demonstrated (Degenkolb et al. 2015).

Few fungal secondary metabolites have been tested for their potential nematicidal activities, and the systematic screening of fungal natural product libraries could therefore reveal the previously unknown nematicidal or antihelmintic activities of known secondary metabolites (Laatsch 2014). However, there are no standardized protocols for the quantitative determination of nematicidal activity, and compounds that are active against model nematodes used in bioactivity assays may not be active against field pests and vice versa (Anderson et al. 1995).

Several issues remain to be resolved before nematophagous fungi can be used for the biocontrol of nematodes. First, many nematophagous fungi are facultative parasites. This is desirable because artificial mass production of propagules is required to achieve a positive cost-benefit ratio, but species and strains that grow well under artificial, saprotrophic conditions may be less inclined to parasitize pest nematodes in the field, and antagonists that capture nematodes efficiently may not establish well in the soil (Cooke 1968). Second, the host range of many nematophagous species is comparatively narrow, as exemplified above for *D. coniospora*. Consequently, parasites of root-knot nematodes may not be able to parasitize eggs (Moosavi and Zare 2012). Third, it is important to note that many nematicidal metabolites produced by fungi are comparatively unstable, including unsaturated fatty acids (compounds **1**, **81**, **82**), oligosporon-type antibiotics (compounds **2**–**16**), and RALs (compounds **24**–**30**, **32**–**50**). It is therefore unlikely that such compounds could be used in their pure forms, although they may play a hitherto unknown role following direct contact between a nematophagous fungus and its nematode hosts (Hellwig et al. 2003, Stadler et al. 2003). Finally, health and safety aspects must be considered before a nematophagous fungus can be registered as an agricultural biocontrol agent, as illustrated by the following two examples.

As stated above, different strains of *P. variotii* (syn. for teleomorph *Byssochlamys spectabilis*) can produce herbicidal, insecticidal, and nematicidal secondary metabolites (compounds **70**–**72**), but some strains also produce viriditoxin, the C-6/C-6′ dehydro dimer of semi-viriditoxin (compound **83**), (Houbraken et al. 2006), which is a mycotoxin in a strict sense (Frisvad et al. 2006). Structurally related compounds such as semi-viriditoxin (compound **63**) and semi-viriditoxic acid (compound **64**) could therefore exhibit similar, undesirable effects towards vertebrates.

Although *P. lilacinum* (syn. *Paecilomyces lilacinus*) is a ubiquitous, saprotrophic soil-borne species, which is regularly found as a pathogen of nematodes and insects, it is also pathogenic to humans and other animals. Some strains of this species cause keratitis, but pathogenic and non-pathogenic strains cannot be distinguished based on their ribosomal RNA internal transcribed spacer or transcriptional enhancer factor 1α sequences (Luangsa-ard et al. 2011). The pronounced heterogeneity of the aggregate species *P. lilacinum* has already led to the description of two additional species, *Purpureocillium lavendulum* (Perdomo et al. 2013) and *Purpureocillium takamizusanense*^15^ (Ban et al. 2015). However, Eberhard et al. (2014) emphasized that *P. lilacinum* (sensu Luangsa-ard) is far more diverse with regard to habitat, geographical distribution, and especially ITS sequence homology. This may ultimately lead to the delimitation of the nematode pathogens from human-pathogenic and other strains. Recently, a fourth official combination, *Purpureocillium atypicolum*, was published by Spatafora et al. (2015). The genus is currently supposed to encompass all purple- to lilac-spored species previously classified in *Nomuraea*, although they have not been validly combined yet. However, many more species are expected to belong to *Purpureocillium* (Gams W, personal communication).

The decline in the availability of chemical measures for efficient nematode control is a challenge that must be met by intensifying the search for environmentally beneficial and cost-efficient alternatives. Nematophagous fungi provide a largely unexploited yet promising reservoir of novel nematicides, which could be used for the biocontrol of phytoparasitic nematodes.

## Footnotes

1 Phylogenetic-based nomenclatural proposals of nematophagous *Ophiocordycipitaceae* (*Hypocreales*) were recently published by Quandt et al. (2014).

2 Erroneously reported as CBS 642.8 by Wu et al. (2013).

3 Liu et al. (1999) hypothesized a biosynthetic origin from (*S*)-(+)-abscisic acid (ABA) by oxidative removal of the two terminal carbon atoms, as the conversion of blumenol A into (*S*)-(+)-ABA has previously been reported by Galbraith and Horn (1973).

4 A biosynthetic pathway starting from tryptophane, glycine, and alanine was proposed (Guo et al. 2011).

5 Compared to the radicicol family, the important mycotoxin zearalenone and its derivatives display the opposite (= l) configuration at C10′. Biosynthetic enzymes are only distantly related, so they may have evolved independently from a common ancestor (Heberling et al. 2014).

6 Originally isolated from *Cylindrocarpon destructans* var. *destructans* (syn. *Cylindrocarpon radicicola*) and thus also widely known as radicicol (Evans and White 1966).

7 Originally isolated from *Pseudeurotium ovalis* (Bloch et al. 1976).

8 For details, see Ayer et al. (1980).

9 Peptaibiotics have comprehensively been reviewed by Degenkolb and Brückner (2008), including comments on non-ribosomal peptide biosynthesis and modes of action.

10 For phylogenetic re-evaluation of the genus *Acremonium*, see Summerbell et al. (2011) and Giraldo et al. (2015).

11 Nematicidal bioactivities have not been reported for any of the 218 *Acremonium*-derived metabolites that are currently listed in AntiBase (Laatsch 2014).

12 With the exception of compounds described in this review, nematicidal bioactivities have not been reported for any of the 181 *Paecilomyces*-derived metabolites that are currently listed in AntiBase (Laatsch 2014).

13 Fungal metabolites containing a nine-membered ring fused to one or two maleic anhydride. A hypothetic scheme for biosynthesis of these compounds was recently published (Szwalbe et al. 2015).

14 The current name of *Paecilomyces lilacinus* is *Purpureocillium lilacinum* (Luangsa-ard et al. 2011). However, it appears that the new name is still rarely used in the biocontrol literature.

15 Hitherto exclusively isolated from cicada species.
